# The relationship between quantitative measures of disc height and disc signal intensity with Pfirrmann score of disc degeneration

**DOI:** 10.1186/s40064-016-2542-5

**Published:** 2016-06-22

**Authors:** Sara Salamat, John Hutchings, Clemens Kwong, John Magnussen, Mark J. Hancock

**Affiliations:** Physical Therapy Department,Tehran university of Medical Sciences, District 12, Enghelab, Pich-e-Shemiran, Tehran, Iran; Faculty of Medicine and Health Sciences, Macquarie University, Talavera Rd North Ryde, Sydney, NSW 2109 Australia; Macquarie Medical Imaging, Macquarie University Hospital, Talavera Rd North Ryde, Sydney, NSW 2109 Australia

**Keywords:** Disc degeneration, Disc signal, Disc height, Reliability, Magnetic resonance imaging, Pfirrmann scores, Validity, Diagnosis, Low back pain, Imaging, Correlation

## Abstract

**Purpose:**

To assess the relationship between quantitative measures of disc height and signal intensity with the Pfirrmann disc degeneration scoring system and to test the inter-rater reliability of the quantitative measures.

**Methods:**

Participants were 76 people who had recently recovered from their last episode of acute low back pain and underwent MRI scan on a single 3T machine. At all 380 lumbar discs, quantitative measures of disc height and signal intensity were made by 2 independent raters and compared to Pfirrmann scores from a single radiologist. For quantitative measures of disc height and signal intensity a “raw” score and 2 adjusted ratios were calculated and the relationship with Pfirrmann scores was assessed. The inter-tester reliability of quantitative measures was also investigated.

**Results:**

There was a strong linear relationship between quantitative disc signal intensity and Pfirrmann scores for grades 1–4, but not for grades 4 and 5. For disc height only, Pfirrmann grade 5 had significantly reduced disc height compared to all other grades. Results were similar regardless of whether raw or adjusted scores were used. Inter-rater reliability for the quantitative measures was excellent (ICC > 0.97).

**Conclusions:**

Quantitative measures of disc signal intensity were strongly related to Pfirrmann scores from grade 1 to 4; however disc height only differentiated between grade 4 and 5 Pfirrmann scores. Using adjusted ratios for quantitative measures of disc height or signal intensity did not significantly alter the relationship with Pfirrmann scores.

## Background

The clinical importance of lumbar disc degeneration as measured on MRI remains controversial and uncertain. This may in part be due to the challenges in accurately and reproducibly measuring disc degeneration. Most studies investigating disc degeneration use a subjective assessment which categorize discs into different levels of degeneration. The most widely used assessment of disc degeneration is the 5-grade classification system of disc degeneration proposed by Pfirrmann et al. (2001). This grading system is primarily based on changes in signal intensity, distinction between nucleus and annulus fibrosis and disc height (Pfirrmann et al. 2001; Niu et al. 2011). The scale lacks sensitivity to change and has only moderate to good reliability (Videman et al. 2006; Borthakur et al. 2011).

To overcome some of these limitations quantitative measures of disc degeneration on MRI have been developed and used. These quantitative measures most commonly include measurement of the signal intensity of nucleus pulposus and the disc height (Videman et al. 2008; Tunset et al. 2013; Watanabe et al. 2007; Niemeläinen et al. 2008). Quantitative measurements are generally reported to have excellent reliability (Videman et al. 2008; Teichtahl et al. 2015) and are more sensitive to change than traditional subjective scales. These quantitative measures may be important in future research investigating the potential relationship between disc degeneration on MRI and current or future low back pain; however, little is known about the validity of these measures and how they relate to the widely used subjective assessments.

To be useful in assessing the clinical importance of MRI findings, measures must be capable of comparing between individuals as well as within individuals over time. It is questionable if quantitative measures of disc degeneration can be validly used for this purpose. For example, it is unclear if it is reasonable to compare quantitative measures of disc height between 2 individuals who have very different overall height. Do quantitative measures of disc height relate to the subjective measures of disc degeneration made on MRI scans which may be influenced by other factors such as adjacent discs and vertebral body height? Similarly quantitative measures of disc signal are commonly adjusted for local cerebrospinal fluid (CSF) to allow for local variations in image intensity both within a single image and between different images (Videman et al. 2008). There has been limited investigation of the validity of these measures between patients or the relationship with subjective measures of disc degeneration.

Therefore, the primary aim of the current study was to assess the relationship between quantitative measures of disc height and disc signal intensity with Pfirrmann scores and to investigate whether different methods of assessing these variables influenced the relationship. We also wished to investigate the inter-rater reliability of the quantitative measures of disc height and disc signal intensity.

## Methods

This study used baseline data from a previous cohort study investigating whether MRI findings predicted time to a recurrence of low back pain in 76 participants who had recently recovered from a previous episode of low back pain (Hancock et al. The Spine journal, accepted July 2015) (Hancock et al. 2015). The study was approved by Macquarie University Human Ethics Committee.

### Participants

Participants were included if they had recovered from a previous episode of acute, non-specific LBP (pain in the area between the 12th rib and buttock crease, with or without leg pain) within the last 3 months. The date of recovery was defined as the 30th consecutive day with pain no greater than 1 on a 0–10 scale. Exclusion criteria included: previous spinal surgery, contraindication to MRI or being unable to complete follow-up electronically for the cohort study.

### MRI examination

All participants underwent a Lumbar Spine MRI scan on a single high field strength system (3.0 Tesla Siemens Verio) with a multichannel phased array spine surface coil. A standardized protocol was used for all participants, which included sagittal fast spin-echo T1 (TR 650 ms, TE 6.3 ms) and T2 (TR 4500 ms, TE 101 ms), sagittal STIR (TR 3800 ms, TE 35 ms, IR 215 ms) and axial T2 (TR 5000 ms, TE 116 ms) scans. All sequences were 4 mm thick with a 1 mm inter-slice space. Sagittal sequences used a 320 mm FOV and axial 200 mm (Hancock et al. 2015).

### MRI measures (Pfirrmann scores)

A single, experienced radiologist rated disc degeneration for all lumbar levels in the participants (380 disc levels in total) based on Pfirrmann’s score (Pfirrmann et al. 2001), using the criteria listed in Table 1. The intra-tester reliability was previously reported to be good (K = 0.86) (Hancock et al. The Spine journal, accepted July 2015) (Hancock et al. 2015). The radiologist was blinded to the quantitative measures.

**Table 1:** Pfirrmann disc degeneration grading system (Pfirrmann et al. 2001)

Score	Structure	Distinction of nucleus and annulus	Signal intensity	Intervertebral disc height
1	Homogeneous, bright white	Clear	Hyperintense, isointense to CSF	Normal
2	Inhomogeneous with or without horizontal bands	Clear	Hyperintense, isointense to CSF	Normal
3	Inhomogeneous, gray	Unclear	Intermediate	Normal to slightly decreased
4	Inhomogeneous, gray to black	Lost	Intermediate to hypointense	Normal to moderately decreased
5	Inhomogeneous, black	Lost	Hypointense	Collapsed disc space

### MRI measures (quantitative measures of disc height and signal intensity)

Quantitative measures of disc height and disc signal intensity were made for each of the 5 lumbar discs in all participants. The measures were made by 2 of the researchers (JH and CK) who were both final year Physiotherapy students and blinded to the participants’ details and Pfirrmann scores. Before starting quantitative measures they were trained by a radiologist (JM) on 2 occasions and practiced for 2 weeks using a highly standardized protocol until good reliability was achieved.

**Fig. 1:**
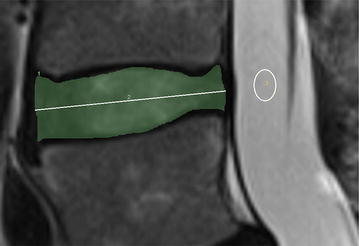
MRI tracing for quantitative measures of disc height and disc signal intensity. The *shaded region* represents the area of the disc. Disc area was defined by using the freehand region of interest measurement tool and tracing around the disc starting along the anterior longitudinal ligament, moving along the superior disc-vertebral interface, posterior longitudinal ligament and finally inferior disc-vertebral interface. Raw disc height was measured by dividing the disc area by length of horizontal line between anterior and posterior boundaries of intervertebral disc. The *elliptical region* represents the cerebrospinal fluid reference sample

Inteleviewer, version 4.3.4, image analysis software was used to take quantitative measures of disc height and disc signal intensity on T2 midsagittal images. We investigated 3 different measures of disc height. These included 1) a raw disc height measure, 2) a ratio adjusted for each person’s height (ratio 1) and 3) a ratio adjusted for height of the vertebral body above the disc (ratio 2). Raw disc height was measured by dividing the disc area by horizontal length (distance between anterior and posterior boundaries of intervertebral disc) in a manner similar to previous studies (Videman et al. 2014). Disc area was defined by using the freehand region of interest measurement tool and tracing around the disc starting along the anterior longitudinal ligament, moving along the superior disc-vertebral interface, posterior longitudinal ligament and finally inferior disc-vertebral interface (Fig. 1). Ratio 1 was calculated by dividing the raw disc height for each vertebral level by the total body height of the participant. Ratio 2 was calculated by dividing the raw disc height by the height of the vertebral body above the disc. The height of the vertebral body above was calculated in a similar manner to disc height.

We also investigated 3 different measures of disc signal intensity. These included (1) a raw disc signal intensity measure (2) a ratio adjusted for brightness of CSF (Battié et al. 1995; Videman et al. 1994) at the same level (ratio 1) and (3) a ratio adjusted for brightest level of CSF at any of the 5 spinal levels (ratio 2). Raw signal intensity was recorded for each disc area as defined above for measurement of disc height. All measurements of signal intensity were made using primary DICOM data, preserving the full dynamic range of the raw data. Ratio 1 was calculated by dividing the raw disc signal intensity for each vertebral level by the signal intensity of the CSF at the adjacent level. Ratio 2 was calculated by dividing the raw disc signal intensity by the signal intensity of the most intense CSF at any of the 5 spinal levels. A clean sample of CSF adjacent to vertebra levels was used as the intra-body reference standard, accept at stenotic levels where it was difficult to obtain a clean sample so the level of the vertebral body above was used.

### Analysis

To investigate the inter-tester reliability of the quantitative measures of disc height and signal intensity we used intra class correlation coefficient (ICC) comparing scores from both assessors across the 380 disc levels.

To investigate the relationship between Pfirrmann scores and each of the quantitative measures of disc height and signal intensity we calculated the mean and SD of each quantitative measure for each Pfirrmann score (1–5), across all 380 discs. We also plotted these to help visual inspection of the relationship. All quantitative measures were based on average scores from the 2 assessors. To statistically test the relationship between Pfirrmann scores and quantitative measures we used one way ANOVA, with (Pfirrmann score as independent variable) and quantitative MRI score as dependent variable. The dependent variable was tested to ensure normality. If the ANOVA was significant we then performed post hoc testing with Tukey’s test, to test paired comparisons between each level of Pfirrmann scores (e.g. 4 vs 5).

To assess the strength of the relationship between Pfirrmann scores and each of the quantitative measures we planned to perform linear regression to determine the explained variance (R^2^), if the visual inspection of the association when plotted suggested a linear relationship.

SPSS software 21 was used for statistical analysis and the level of significance was set at p < 0.05.

## Results

Between September 2012 and April 2013, 76 people were enrolled in the study. The characteristics of included participants are presented in Table 2. Participants mean age was 45 and all were recently free from low back pain, having recovered from an episode of low back pain within the previous 3 months.

**Table 2:** Baseline characteristics

	Participant s (N = 76)
Male gender, n (%)	46 (60.5)
Age, mean (SD)	45 (13)
Height (cm), mean (SD)	171.38 (9.6)
Weight (kg), mean (SD)	79.63 (18.78)
BMI, mean (SD)	26.9 (5.1)
Previous episodes of low back pain median, (IQR)	2.5 (1–7.8)

### Reliability of quantitative measures

The reliability values were excellent for both disc height and signal intensity measures, regardless of whether the raw scores were used or either of the 2 ratios. ICC ranged from 0.97 (95 % CI 0.96–0.97) to 0.98 (95 % CI 0.97–0.98) for signal intensity and 0.96 (95 % CI 0.953–0.9769) to 0.97 (95 % CI 0.95–0.97) for disc height.

### Relationship between quantitative measures of disc height and Pfirrmann score

**Fig. 2:**
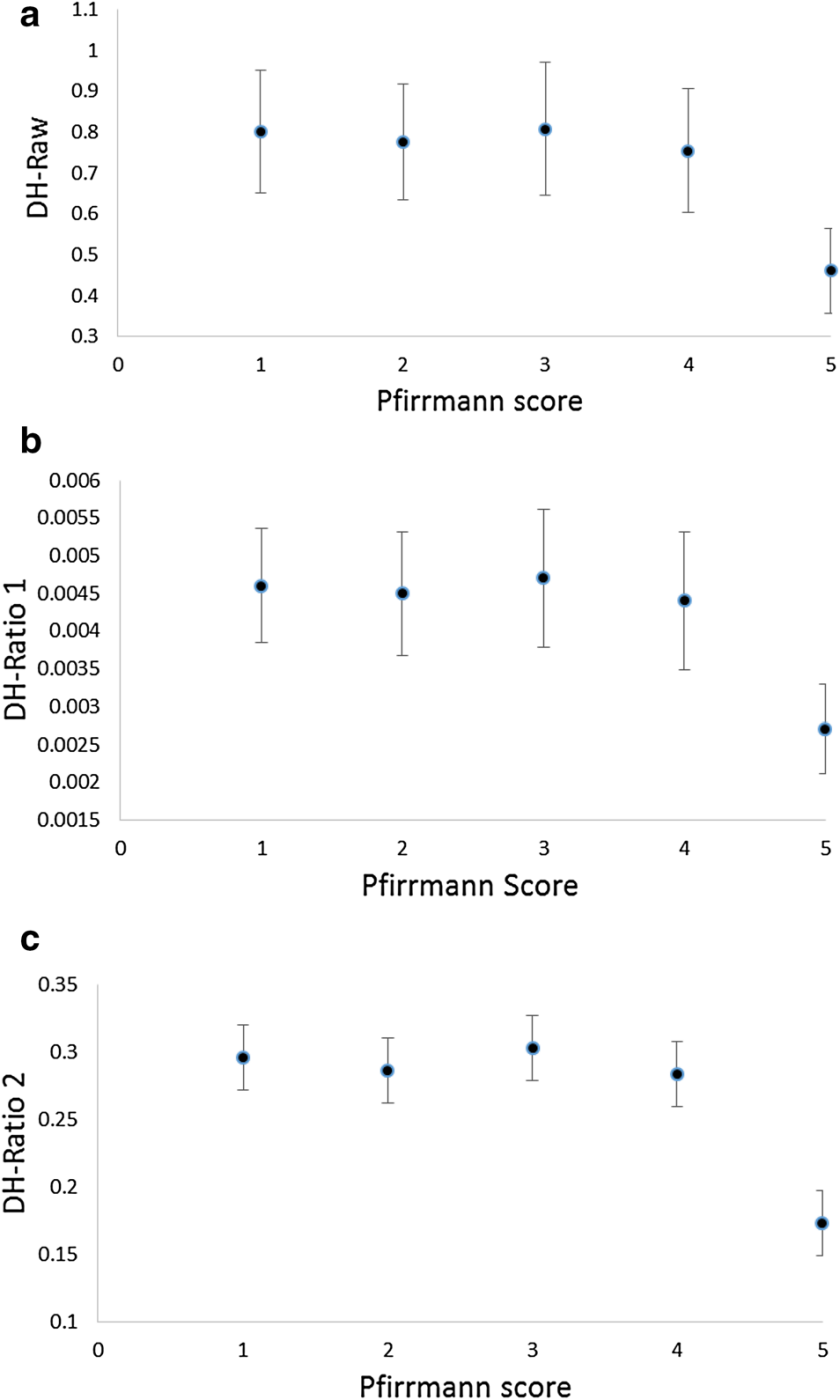
Relationship between quantitative disc height with Pfirrmann scores. **a** Relationship between raw disc height and Pfirrman scores. **b** Relationship between disc height ratio 1 and Pfirrman scores. **c** Relationship between raw disc height ratio 2 and Pfirrman scores. DH = disc height; raw disc height was measured by dividing the disc area by length of horizontal line between anterior and posterior boundaries of intervertebral disc; disc height ratio 1 was calculated by dividing raw disc height by the person’s height (cm); disc height ratio 2 was calculated by dividing raw disc height by height of the vertebral body above the disc (cm). *Data points* represent the mean and *error bars* represent SD

The relationship between the 3 quantitative measures of disc height (raw score, ratio 1 and ratio 2) can be seen in Table 3 and Fig. 2. The ANOVA suggests that there is a relationship (p < 0.001); however, the relationship is clearly not linear. There is no relationship between quantitative disc height measures and Pfirrmann scores from grades 1 to 4 (Table 3; Fig. 2); however, disc levels with Pfirrmann score of grade 5 have significantly lower quantitative disc height scores than discs with grades 1–4 Pfirrmann scores. These findings were consistent regardless of whether we used raw disc height scores or ratio 1 or 2. Due to the non-linear relationship we did not explore the explained variance (R^2^) using linear regression.

**Table 3:** Relationship between quantitative disc height and signal intensity with Pfirrmann’s score

	Pfirrmann 1 N = 24	Pfirrmann 2 N = 165	Pfirrmann 3 N = 96	Pfirrmann 4 N = 82	Pfirrmann 5 N = 13	p value Significant pairwise comparisons (p < 0.01)
DH_Raw (cm)	0.80 (0.14)	0.77 (0.14)	0.81 (0.16)	0.75 (0.15)	0.46 (0.10)	Pfirrmann 5 compared to all other Pfirrmann levels
DH ratio 1	0.0046 (0.0007)	0.0045 (0.0008)	0.0047 (0.0009)	0.0044 (0.0009)	0.0027 (0.0005)	Pfirrmann 5 compared to all other Pfirrmann levels
DH ratio 2	0.29 (0.05)	0.28 (0.05)	0.30 (0.06)	0.28 (0.06)	0.17 (0.03)	Pfirrmann 5 compared to all other Pfirrmann levels
SI-Raw	245.7 (58.3)	208.4 (52.7)	138.55 (34.0)	98.7 (22.7)	76.2 (22.7)	All comparisons except Pfirrmann 4 compared to 5
SI-ratio1	0.30 (0.10)	0.22 (0.06)	0.14 (0.03)	0.11 (0.02)	0.08 (0.03)	All comparisons except Pfirrmann 4 compared to 5
SI_ratio2	0.25 (0.07)	0.19 (0.05)	0.13 (0.03)	0.09 (0.03)	0.07 (0.03)	All comparisons except Pfirrmann 4 compared to 5

All values are mean (SD). DH = disc height; disc height ratio 1 was calculated by dividing by the persons height (cm); disc height ratio 2 was calculated by dividing by height of the vertebral body above the disc (cm); SI = Signal intensity; Signal intensity ratio 1 was calculated by dividing by the cerebrospinal fluid brightness at the same spinal level; signal intensity ratio 2 was calculated by dividing by the brightest cerebrospinal fluid at any spinal level

### Relationship between quantitative measures of disc signal intensity and Pfirrmann score

**Fig. 3:**
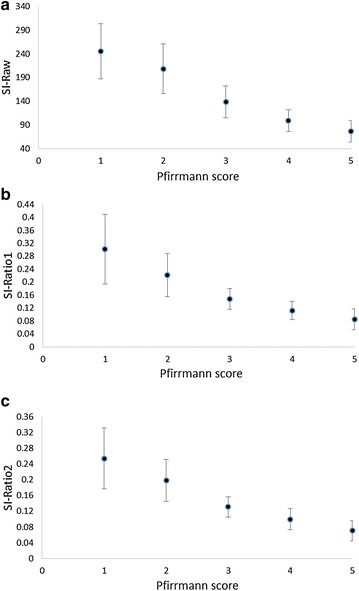
Relationship between disc signal intensity with Pfirrmann scores. **a** Relationship between raw disc signal intensity and Pfirrman scores. **b** Relationship between disc signal intensity ratio 1 and Pfirrman scores. **c** Relationship between disc signal intensity ratio 2 and Pfirrman scores. SI = signal intensity; Signal intensity ratio 1 was calculated by dividing the raw signal intensity value by the intensity value of the cerebrospinal fluid reference at the same spinal level; signal intensity ratio 2 was calculated by dividing by the raw signal intensity value by the intensity value of the brightest cerebrospinal reference at any spinal level. *Data points* represent the mean and *error bars* represent SD

The relationship between the 3 quantitative measures of disc signal intensity (raw score, ratio 1 and ratio 2) can be seen in Table 3 and Fig. 3. The ANOVA suggests that there is a relationship (p < 0.001), which appears to be linear between quantitative signal intensity measures and Pfirrmann scores grade 1–4 (Table 3; Fig. 3). Paired comparisons found no significant difference in signal intensity between discs with a Pfirrmann score of 5 compared to 4. These findings were consistent regardless of whether we used raw signal intensity scores or ratio 1 or 2. Due to the linear relationship we explored the explained variance (R^2^) using linear regression. R^2^ for raw signal intensity, ratio 1 and 2 was 0.57 (p < 0.001), 0.49 (p < 0.001) and 0.55 (p < 0.001) respectively.

## Discussion

### Main findings

Our results showed a linear association between signal intensity and Pfirrmann scores. The signal intensity decreases with the increasing extent of disc degeneration according to Pfirrmann scores, except for between grade 4 and 5 where the decrease was not statistically significant. In contrast quantitative measures of disc height were only statistically reduced for the most degenerative discs (Pfirrmann grade 5). Whether we used raw or adjusted measures of disc signal intensity or disc height did not substantially change the relationship between disc height or signal intensity with Pfirrmann scores. The quantitative measures of disc signal intensity and height had excellent inter-tester reliability.

### Strengths and weaknesses

Our study has several strengths that increase the validity of the findings. We recruited a homogeneous sample of patients who had recently recovered from acute low back pain and followed a strict imaging protocol for all participants using a single high field strength system. Subjective and quantitative MRI measures were conducted according to strict criteria and there was no missing data. We investigated both a “raw” measure and 2 adjusted measures of both disc height and signal intensity enabling us to further explore the ability of different quantitative measures to compare between individuals. A weakness of our study is that we had a relatively small number of discs with a grade 5 Pfirrmann score so we have less power in comparing this group to the other Pfirrmann grades. We did not standardize the time of day at which imaging was performed and this may have a small impact on disc height or signal intensity. Also all quantitative measures were done by 2 master students with limited prior experience; however, the results demonstrated excellent reliability.

An alternative approach to measuring disc signal intensity, which was not used in the current study, is T2 relaxation time mapping (Watanabe et al. 2007). This approach may be more accurate and does not require normalisation to CSF as performed in the current study; however, this approach requires a longer scanning time and a more complex and time consuming analysis making it less practical for routine clinical use.

### Comparison to previous studies

To our knowledge this is the first study to compare different quantitative measures of both signal intensity and disc height with Pfirrmann scores. Niu et al. (2011) compared 2 quantitative MRI imaging tools (apparent coefficient diffusion and T2 signal intensity) with each other and Pfirrmann scores in people with low back pain and healthy subjects. They concluded T2 intensity is a sensitive method for detecting early stages of disc degeneration. This is consistent with our finding that signal intensity has a strong association with Pfirrmann grades 1–4. Luoma et al. (2001) quantitatively measured disc height (ant and post height) and signal intensity in 109 men working in 3 different occupational roles, using T2 weighted MRI. Similar to our findings they question the validity of disc height as an early measure of disc degeneration. Teichtahl et al. (2015) compared quantitative measures of disc height with Pfirrmann scores in 72 community based individuals. They found small reductions in disc height from grade 2 to grade 4 Pfirrmann scores and then a large reduction with grade 5 Pfirrmann scores. Jarman et aI. (2015) reported a disc height index was associated with Pfirrmann scores especially at the more severe levels of disc degeneration. These studies are somewhat different to our findings in that they did find an association between disc height and Pfirrmann scores even at lower grades; however, the differences were small and most obvious at Pfirrmann grade 5 as per our findings.

Several studies have tested the reliability of quantitative measures of disc height and signal intensity (Fan et al. 2012; Hon et al. 2014; Pfirrmann et al. 2006) and reported high reliability. Our study shows excellent inter-tester reliability can be achieved in raters who are not radiologists but undergo a training program and follow a strict protocol. This has important implications for future studies.

### Meaning of the study/implications

Our study suggests that quantitative measures of disc height and signal intensity must be used carefully to assess changes both within and between individuals with back pain. While the measures are reliable and sensitive to small changes our findings suggest signal intensity is likely to be sensitive to early to moderate disc degeneration, while disc height measures are only sensitive to end stage degeneration. The strong relationship between quantitative measures of disc signal intensity and Pfirrmann scores, suggests signal intensity could be used instead of Pfirrmann scores in studies where the increased sensitivity to change and reliability of the quantitative measures is important.

An interesting finding from our study was that the relationship between signal intensity and Pfirrmann’s was similar regardless of whether we used raw values or ratios. This suggests that raw scores may be acceptable for comparing between individuals. In particular we note that the relationship between disc height and Pfirrmann scores was similar regardless of whether we used ratios that allowed for a person’s height or not. Similarly the common practice of adjusting signal intensity by the CSF at a similar disc level did not influence the relationship with Pfirrmann scores. Earlier MRI imaging may have had greater spatially dependent inhomogeneity of signal intensity in the FOV than was present in our study. If however, significant spatially dependent inhomogeneity did exist it would seem logical that using some reference to allow for this would still be important to compare between discs and different scans. In our study we used a single 3T scanner and followed a strict protocol. Where this is not the case the use of CSF reference may be more important. We would expect the findings to be similar on a 1.5 T scanner, allowing for differences in spatial resolution and the intrinsic reduction in signal to noise ratio. Internal normalisation is an effective technique, independent of field strength.

## Conclusions

Quantitative measures of disc signal intensity are strongly related to Pfirrmann scores from grade 1 to 4; however, quantitative measures of disc height only differentiate between grade 4 and 5 Pfirrmann scores. Using adjusted values for quantitative measures of disc height or signal intensity did not significantly alter the relationship with Pfirrmann scores; however, this may need to be investigated for multicenter or multi-scanner studies.
